# Dorsal Root Ganglion Stimulation for Thoracic Neuralgia: A Report of Six Cases

**DOI:** 10.7759/cureus.4615

**Published:** 2019-05-07

**Authors:** Casey L Anthony, Muhibullah S Tora, Jessica N Bentley, Pavlos Texakalidis, Nicholas M Boulis

**Affiliations:** 1 Neurosurgery, Emory University School of Medicine, Atlanta, USA; 2 Neurosurgery, Emory University School of Medicine, Atlanta, USA; 3 Neurosurgery, University of Alabama at Birmingham, Birmingham, USA; 4 Neurosurgery, Emory University Hospital, Atlanta, USA

**Keywords:** dorsal root ganglion, stimulation, thoracic, thorax, pain, thoracic neuralgia, neuropathic pain

## Abstract

Objective: Thoracic neuralgia (TN) is a chronic pain syndrome that can be refractory to pharmacologic intervention and management by pain specialists. Dorsal root ganglion (DRG) stimulation has shown promise as a targeted and effective modality compared to traditional therapies for several indications but has not yet been applied in the thoracic region. This study aims to report the outcomes of off-label thoracic DRG stimulation in patients with refractory TN.

Methods: A retrospective chart review was performed at Emory University Hospital for patients who underwent thoracic DRG stimulation in a two-year period. Relevant outcomes for safety and efficacy were evaluated.

Results: Six patients were identified that underwent thoracic DRG stimulation for various etiologies of TN, including post-mastectomy, post-herpetic, and post-abdominoplasty neuralgia. All patients initially underwent trial DRG stimulation with a mean pre-operative visual analogue scale (VAS) (0-10) of 6.8 ± 1.6 (range: 4-8). Four of six patients (67%) were non-responders and did not pursue permanent implantation; two experienced pain with stimulation during the trial, and two patients experienced no significant benefit. In addition, all three patients with post-herpetic neuralgia did not respond to treatment. Two of six patients (33%) responded well to stimulation, elected to receive permanent leads, and reported significant pain relief with VAS scores of 0/10 and 1/10, and 100% reduction in morphine equivalent use. Complications included lead migration and need to reset stimulator programming.

Conclusions: DRG stimulation may be an effective therapy for patients experiencing chronic TN as a result of peripheral nerve injury; however, post-herpetic neuralgia may be unresponsive to this treatment. Future prospective studies are warranted to evaluate the feasibility of this procedure in patients with refractory TN.

## Introduction

Thoracic neuralgia (TN) is a chronic pain syndrome with various causes, including thoracotomy, mastectomy, and herpes zoster infection. While the true incidence is difficult to define, 3%-22% of pain clinic patients are referred for thoracic pain, and these etiologies are associated with a high risk of severe chronic postoperative or post-infection pain [1]. For instance, debilitating post-thoracotomy pain syndrome affects 50% of patients, and 66% seek treatment for pain [2]. Even though chronic pain syndromes are prevalent and debilitating in many cases, the therapeutic armamentarium is limited, and even management by pain specialists can remain ineffective. Multiple treatments are routinely utilized to manage neuropathic pain, including pharmacologic treatment, physical therapy, transcutaneous electrical nerve stimulation, intercostal nerve blocks, and neuromodulating agents. For medically refractory patients, spinal cord stimulation (SCS) is traditionally offered. Pain remission success rates range from 50%-75% depending on the indication, with the most successful outcomes for failed back surgery syndrome and complex regional pain syndrome (CRPS); however, thoracic stimulation is particularly difficult compared to cervical and lumbar regions due to challenges in targeting tight dermatome bands with subsequent effects on surrounding neural structures and has been effective in select cases [3-5]. These treatments are successful in managing a majority of patients with chronic pain, but many cases are still uncontrolled even with SCS. This is attributed to a variety of factors, including positional variations with stimulation, the shunting of energy to cerebrospinal fluid, lack of precision, and segmentation of spinal sensory input [5].

In the last ten years, dorsal root ganglion (DRG) stimulation has become available for treating neuralgia and has shown promise to provide a more targeted and effective modality compared to SCS for pain relief. The DRG resides in the foramen in close proximity to the pedicle. It plays a key role in the development of neuropathic pain, so stimulation can allow a more focused, and effective therapy. Studies have shown that electrical stimulation of the DRG modulates neuropathic pain signals with root specificity and minimal effect with postural changes [6-8]. A trial comparing DRG and spinal cord stimulation for CRPS showed a greater treatment success, quality of life, and psychological disposition in the DRG arm than in the SCS arm with patients receiving >50% pain relief. In addition, subjects reported less postural variation and extraneous stimulation in nonpainful areas [5]. While DRG stimulation has gained traction for cervical and lumbar pain syndromes, there are only a few case reports of thoracic DRG stimulation with no ongoing clinical trials. (Poster: Ali R, Epstein L, Khelemsky Y. Successful Treatment of Chronic Post Thoracotomy Pain Syndrome with Dorsal Root Ganglion (DRG) Stimulation. 20th Annual Meeting of the North American Neuromodulation Society; 2017), (Poster: Justiz R, Smith N. Thoracic DRG Stimulation for Chronic Abdominal Pain Due to Hereditary Pancreatitis. 20th Annual Meeting of the North American Neuromodulation Society; 2017). This study aims to report the outcomes of thoracic-level DRG stimulation in six patients with chronic thoracic pain syndromes of various indications.

## Materials and methods

Operative technique

Abbot Axium Neurostimulator System SlimTip (MN10450-50A, St. Jude Medical, St. Paul, MN, USA) and Patient Programmer (MN10600-02, St. Jude Medical, St. Paul, MN, USA) were used for these procedures. Patients are brought to the operative suite and prepared in a standard fashion in a prone position. Intraoperative fluoroscopy is used to identify the appropriate vertebral body and spinous process and the overlying skin is marked with indelible ink. A stab incision is made at this site with a #15-blade. A 14-gauge Tuohy needle is inserted towards the pedicle of the appropriate spinal level and side using intraoperative fluoroscopy for guidance. The needle is advanced to the inferior margin of the lamina into the intralaminar space, then through the ligamentum flavum into the epidural space. A guidewire is passed through the needle into the epidural space adjacent to the pedicle to confirm proper location. Once this is confirmed, the guidewire is then removed and the introducer cannula is advanced into the epidural space. The introducer is maneuvered inferior to the pedicle. The electrode is then advanced such that the four electrode contacts are positioned within and beyond the pedicle, with the most proximal contact no more medial than the inner border of the pedicle. The introducer is retracted over the wire, and the wire is advanced to form a loop superiorly in the canal to serve as an anchor. The introducer and Tuohy needle are then removed. This is repeated for each of the levels indicated. For trial electrode placement, the distal wires exiting from the skin are connected to an external stimulator. Patients are admitted for observation, then discharged for the trial period lasting seven to 14 days. After a successful trial, a subcutaneous pocket was used for implantation of the implanted pulse generator (IPG) at the buttock or flank.

Chart review

A retrospective chart review was performed to identify all patients at the Emory University Hospital who underwent thoracic DRG stimulation for thoracic neuralgia over a two-year period. Demographic variables were collected, including indication, comorbidities, age, and prior treatments. Pre- and post-operative visual analogue scale (VAS) pain scores, post-operative complications, and narcotic medication doses were also collected. Narcotic medications were converted to morphine equivalents (ME) to provide consistency. Data are represented with descriptive statistics.

Statistical analysis

Categorical variables were summarized as frequencies and percentages, while continuous variables were reported as means, standard deviations (SD) and range using Microsoft Excel. Box plots were used to graphically display continuous variables and the non-parametric Wilcoxon sign rank test was used to compare pre- and post-operative VAS scores using Stata version 14 (Stata Corporation, College Station, TX, USA).

## Results

Six patients were identified who underwent thoracic DRG stimulation for indications including post-mastectomy, post-herpetic, and post-abdominoplasty neuralgia (Table 1).

**Table 1:** Summary of Six Cases Receiving Thoracic Level DRG Stimulation DRG – dorsal root ganglion, F – Female, M – Male, VAS – visual analogue scale, TPI - trigger point injection; TENS - transcutaneous electrical nerve stimulation, NA – Not available, SCS – spinal cord stimulation, PT – physical therapy.

Case	Age (Years)	Sex	Etiology of Thoracic Neuralgia	Duration of Symptoms (Years)	Distribution of Pain	Stimulator Status	Pre / Post Operative VAS	Pre / Post Operative Morphine Equivalent Use	Previous Therapies	Complications/ Reason for Removal
1	42	F	Abdominoplasty	1	Left: T12-L2	Permanent	4 / 0	30 / 0	L1-L2 nerve block, Femoral nerve block, Cutaneous nerve block	Lead migration requiring wire replacement 1 year post-operatively
2	55	F	Bilateral Mastectomy	3	Bilateral: T4-T7	Permanent	8 / 1	25 / 0	Intercostal nerve block, TPI	Programming reset
3	46	M	Herpetic neuralgia	3	Right: T12-L2	Removed	6 / 6	80 / NA	Epidural steroid injection, Ketamine injection, SCS	Pain caused by leads
4	72	F	Mastectomy	3	Left: T2-T4	Removed	7 / 8	1200 / NA	Thoracic facet injection, Intercostal nerve block, TENS, PT, SCS	Pain caused by leads
5	68	M	Herpetic Neuralgia	4	Left: T4-T7	Removed	8 / 8.5	80 / NA	Nerve block, SCS	No pain improvement
6	76	M	Herpetic Neuralgia	7	Left: T1-T3	Removed	8 / 5.5	40 / NA	SCS	Minimal pain improvement

**Figure 1:**
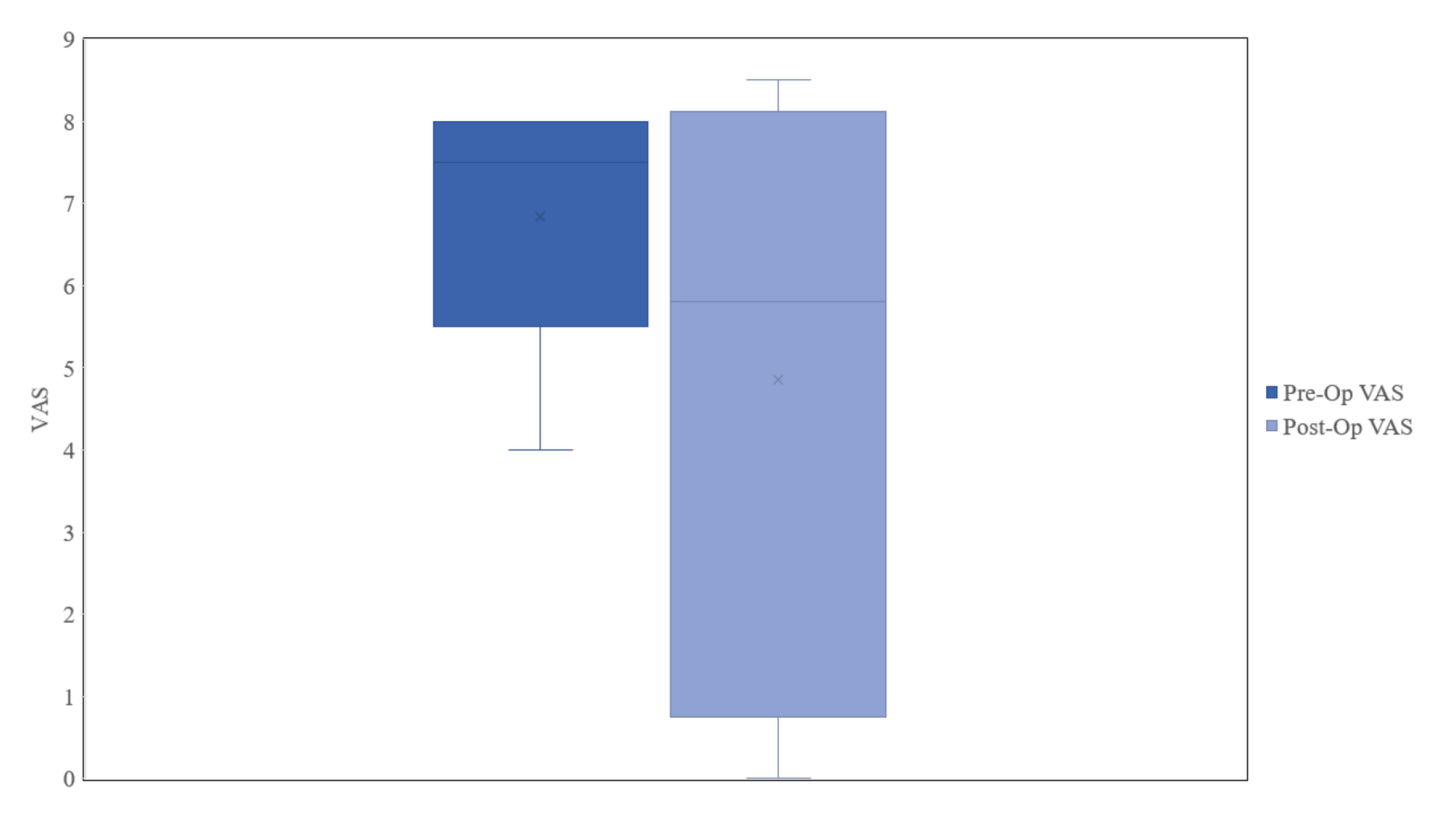
Pre- and Post-Operative Visual Analogue Pain Scores for All Patients VAS – visual analogue scale, Pre-Op – pre-operative, Post-Op – post-operative.

All patients received trial DRG stimulation with a mean pre-operative VAS of 6.8 ± 1.6 and range of 4-8. Mean post-operative VAS score was 4.9 ± 3.6 showing no statistically significant pain reduction as compared to the pre-operative VAS score baseline (p=0.58) (Figure 1).

Two patients received a positive stimulation trial with >90% improvement in pain and proceeded with permanent lead implantation. VAS score was markedly reduced during the eight-month follow-up; however, statistical significance was not reached due to the very small sample size (p=0.17).

The first patient with permanent stimulator implantation had post-abdominoplasty pain in the lower left extremity and groin that began six weeks post-operatively and continued for one year before the stimulation trial. The patient reported a VAS change from 4 to 0/10 after trial stimulation, and permanent leads were placed T12-L2 on the left side with similar pain reduction at eight months follow-up. Following permanent placement, there was a 100% reduction in ME, from 30 to 0. The only reported complication was the migration of leads causing pain, which was relieved by realignment. The second patient with permanent stimulator implantation had post-bilateral mastectomy pain around the ribcage that developed eight months post-operatively and underwent trial stimulation three years later. The patient reported a VAS improvement from 8 to 1/10, and permanent leads were placed T4-T7 on the right and left sides. After permanent implants were placed, similar pain relief was reported at two weeks follow-up. The patient reported acute pain at the electrode site, which was relieved by program resetting. There was a 100% reduction in prescribed ME, from 25 to 0 ME.

Four patients elected not to receive permanent lead implants after trial stimulation. One patient with severe right-sided flank pain due to post-herpetic neuralgia received trial stimulation T12-L2 on the right side three years after onset. The patient reported some initial pain relief, but the leads were removed 14 days after placement due to subsequent pain from the leads. A patient with chronic pain on the left chest and back for four years post-mastectomy received trial stimulation T2-T4 on the left side. Electrodes were eventually removed after 12 days due to intense pain from the leads. Two post-herpetic neuralgia patients experiencing burning pain on the chest for four to seven years received trial stimulation at T4-T7 or T1-T3 on the left side. Both reported no pain improvement with the trial and elected to have the leads removed and not pursue permanent implantation.

## Discussion

The current study demonstrates that 33% of patients responded well to DRG stimulation, with notably thorough pain relief, 100% reduction in ME use, and minimal complications at one to 1.5 years follow-up. Both responders had developed TN due to a history of thoracic surgical intervention, while the etiology of TN of three of the four non-responders was post-herpetic. Of the 67% of non-responders, two experienced pain with trial stimulation, while one experienced transient pain relief followed by no significant benefit based on their VAS scores. Given that thoracic neuralgia due to trauma or iatrogenic injury has been historically difficult to treat [9], DRG stimulation may be of particular interest in this patient population as an option for patients with recalcitrant pain.

Failure of stimulation to provide pain relief could be due to a number of different causes. Because thoracic DRG stimulation is still a new avenue for treatment, lead programming and positioning are not standardized, and improper placement could lead to unchanged or increased pain. In addition, patient willingness to make adjustments to find the best position and setting while in pain may limit the opportunity to optimize the settings. These patients could also represent a population not responsive to this approach. Notably, patients with herpetic neuralgia did not benefit from treatment, while both successful implants were with patients with post-operative pain. Many neuromodulatory strategies for herpetic neuralgia fail because the underlying source of pain in herpetic neuralgia is still unclear and likely multifaceted involving both central and peripheral neurons and neuroplasticity in the dorsal horn [10]. Time from onset to intervention could also influence the likelihood of relief from stimulation. Indication, time from onset, and comorbidities could all contribute to a patient’s response to this therapy.

While this study provides insight into the potential use for DRG stimulation in TN, there are a number of limitations. This was a single-center analysis analyzing outcomes from one provider and approach. While this may help limit discrepancies in methodology, patient environments and previous treatments were not standardized. In addition, since this was a retrospective report of six cases, data relied on accurate patient information and record keeping. Lastly, this was a limited patient pool with only six individuals who met the criteria for the analysis.

## Conclusions

This report of six cases demonstrates the potential success of DRG stimulation as an effective therapy for medically refractory patients experiencing chronic TN. DRG stimulation may be efficacious in patients with thoracic neuralgia due to post-surgical iatrogenic injury. Our cases of post-herpetic neuralgia did not experience pain relief from stimulation. Further studies are warranted to determine the safety and efficacy of this approach and identify accurate patient selection criteria for this treatment strategy.
